# The E-Twenty-Six Family in Hepatocellular Carcinoma: Moving into the Spotlight

**DOI:** 10.3389/fonc.2020.620352

**Published:** 2021-01-27

**Authors:** Tongyue Zhang, Danfei Liu, Yijun Wang, Mengyu Sun, Limin Xia

**Affiliations:** Hubei Key Laboratory of Hepato-Pancreato-Biliary Diseases, Department of Gastroenterology, Institute of Liver and Gastrointestinal Diseases, Tongji Hospital of Tongji Medical College, Huazhong University of Science and Technology, Wuhan, China

**Keywords:** E-twenty-six transcription factor, hepatocellular carcinoma, metastasis, drug resistance, therapy

## Abstract

Hepatocellular carcinoma (HCC) is a major cause of morbidity and mortality worldwide. Although therapeutic strategies have recently advanced, tumor metastasis and drug resistance continue to pose challenges in the treatment of HCC. Therefore, new molecular targets are needed to develop novel therapeutic strategies for this cancer. E-twenty-six (ETS) transcription family has been implicated in human malignancies pathogenesis and progression, including leukemia, Ewing sarcoma, gastrointestinal stromal tumors. Recently, increasing studies have expanded its great potential as functional players in other cancers, including HCC. This review focuses primarily on the key functions and molecular mechanisms of ETS factors in HCC. Elucidating these molecular details may provide novel potential therapeutic strategies for cancers.

## Introduction

Hepatocellular carcinoma (HCC) is a major cause of cancer-related death worldwide, placing a heavy burden on health systems. Although new advances in diagnostic methods and treatment over recent decades, the five-year survival rate of advanced HCC remains low mainly due to its characteristics of frequent metastasis and high drug resistance (1, 2). HCC has high molecular heterogeneity, and its development is a multi-stage biological process accompanying the accumulation of molecular alterations (3). Therefore, it is critically important for us to gain a more comprehensive and profound understanding of regulatory mechanisms in HCC.

The E-twenty-six (ETS) family of transcription factors, evolutionarily conserved and ubiquitously distributed throughout the metazoan lineage, were firstly discovered in the avian leukemia retrovirus E26 (4). Later, ETS1 and ETS2, the human homolog of virus-ETS, were identified on chromosome 11 and 21, respectively (5). To date, 12 subfamilies with 28 family members have been described in humans (6, 7). ETS transcription factors govern the diverse developmental process and vary from embryonic development to adult cells (8, 9). Further, many studies have focused on aberrant expression of the ETS family in diverse human malignancies. Mutations of ETV6 are frequently observed in hematopoietic diseases (10, 11). Besides, gene fusions or gene rearrangements of ETS factors are common driving events in Ewing’s sarcoma and prostate cancer (12, 13). The TMPRSS2/ERG rearrangement drives aberrant expression of ERG in 50% of prostate tumors (14). Moreover, research on targeting ETS fusions is in progress. Also, deregulation of ETS genes affected by inappropriate expression can occur in some other cancers, including breast, liver, colorectal, pancreatic cancer, and melanomas, which may involve different mechanisms and targeting strategies (6, 15–17). This review aims to conclude the roles of ETS family and discuss the regulatory mechanisms and possible therapeutic strategies involving HCC.

## Basic Biology of E-Twenty-Six Family

ETS family is one of the largest transcription families and is sub-classified by their sequence homology and different domains. All the members share a ~85-amino-acid conserved DNA-binding domain, known as the ETS domain, the family defining characteristic (7). It contains a stable winged helix-turn-helix structure (wHTH), recognizing a GGAA/T core motif. ETS factors bind to the genes regulatory regions to direct their expression when helix 3 inserts into the DNA backbone (18, 19). A subset of human ETS factors (11 of 28) is characterized by a pointed (PNT) domain comprising a 65~85-amino-acid region. It performs various functions, such as mediating protein-protein interaction and transcriptional regulation (20, 21). Through rigid body interaction, it plays a vital part in facilitating efficient phosphorylation of ETS1 by ERK2 (22). Moreover, the transactivation domain (TAD) is the key to activate the activity of transcription factors. ERK2 could phosphorylate TAD of ELK1 at multiple sites. The phosphorylation rates at 8 TAD sites are significantly different, which could either promote or inhibit transcriptional activation, thus ensuring a self-limiting response of ELK1 to ERK activation (23).

Different signal-transduction pathways and protein partners could modulate ETS factors. Stimulated by multiple growth factors, ETS factors are upregulated and activated by several signaling pathways, including the Ras/MAPK pathway and PI3K/AKT pathway. Recruiting CBP, the general coactivator of ETS1, the Ras/MAPK pathway could enhance the expression of ETS1 target genes (21, 24). Moreover, ETS factors could cooperate with AP-1 factors (JUN and FOS) whose motifs are close to the ETS binding sequence (13). Regulated by ETS factors, ETS/AP-1 enhancers could mediate promoters’ response to the Ras/ERK pathway, thus promoting ETS1’s own expression (25). And increasingly, a variety of other cooperative partners have been studied. For example, recent studies have found that ETS factors could cooperate with the FOX family as FOX-ETS pairs *via* DNA shape readout. R409 of ETS1 is vital for FOXO1-ETS1 interaction (26).

Although there are sequence similarity and functional redundancy among members, ETS proteins perform their distinct roles depending on the different contexts and regulatory mechanisms. ETS family has been related to various biological processes, such as cell migration, vasculogenesis, hematopoiesis, and homeostasis of epithelial cells. The function of ETS1 in regulating cell mobility is evolutionarily conserved. The mutation of ETS homolog in Drosophila reduces cell migratory potential; upregulation of ETS1 promotes cell migration of human vein endothelial cells (27, 28). ETV2, a master regulator of vasculogenesis, is dispensable for mesodermal lineage development (29). Transient re-expression of ETV2 could reset adult endothelial cells into an embryonic-like stage on which these cells are capable of vascularizing tissues for organ development (30). Several ETS factors, including ETS1, FLI1, PU.1, and SPIB, could control differentiation, localization, and homeostasis of B cells by regulating BCR signaling genes, BCL2 family, and positioning *via* a transcriptional network (31). Furthermore, modulating cell homeostasis is another important function of ETS proteins. In the liver, ERG promotes homeostasis of epithelial cells *via* controlling TGFβ-SMAD signaling. Ablation of ERG in mice leads to endothelial-to-mesenchymal transition and liver fibrosis (32). In addition, ETS1 regulates the antifibrotic properties of hepatic stellate cells (33). These studies suggest that some ETS factors play a protective role in the normal liver. However, when expressed in cancer, they display different abilities.

## The Expression and Clinical Significance of E-Twenty-Six Family in HCC

The aberrant expression of ETS factors is frequently observed in HCC, which is shown in **Table 1**. Of note, the expression of ETS1 in HCC tissues is higher than that in normal liver, whereas it is lower than that in noncancerous lesions (48). Besides, in terms of differently differentiated HCC tissues, the proportion of ETS1 expression is the highest in poorly differentiated HCC samples (49). These suggest a special character of ETS1 in tumorigenesis. Importantly, some ETS factors’ aberrant expression is associated with the clinicopathological characteristics and survival rate of HCC patients. Low PDEF expression was detected in 83.6% HCC patients and was related to larger tumor volume and more frequent microvascular invasion (39). The elevated expression of ELF3 has a positive correlation with larger tumor size and poor prognosis. Besides, it is an independent risk factor for survival of HCC patients (38). Additionally, the time to progression and overall survival is worse in HCC patients receiving sorafenib harboring high ETS1 level than those showing low ETS1 expression (50). These findings suggest that ETS factors might become potential prognostic markers for HCC patients, which would help stratify the patients and evaluate the therapeutic effect.

**Table 1:** The expression of the ETS transcription factors in HCC and their association with clinical-pathological features and patients’ survival.

ETS family	Subcellular localization	Relative Expression level	Correlation with	Prognostic values (H vs L)	Number of patients	Reference
ETS1	Cytoplasm	Low	N.A.	N.A.	29	(34)
ETS1	Nucleus	High	N.A.	N.A.	34	(35)
ETS1	N.A.	High	N.A.	Poor (OS and TTP)	52	(36)
FLI1	N.A.	High	Distant metastasis	N.A.	50	(37)
ELF3	N.A.	High	Tumor size	Poor (OS and DFS)	106	(38)
PDEF	N.A.	Low	Tumor size, microvascular invasion	Good (OS and TTR)	400	(39)
PU.1	N.A.	Low	N.A.	N.A.	38	(40)
ELK1	Nucleus	High	N.A.	N.A.	50	(41)
ELK4	Nucleus	High	N.A.	Good (OS)	278	(42)
ETV4	N.A.	High	N.A.	N.A.	11	(43)
ETV6	N.A.	High	N.A.	N.A.	16	(44)
GABPα	N.A.	Low	Tumor grade, Distant metastasis	Good (OS)	71	(45)
EHF	Cytoplasm	Low	Vascular and capsular invasion Tumor number	Good (OS and DFS)	94	(46)
ELF3	Cytoplasm	High	N.A.	N.A.	5	(47)

OS, overall survival; TTP, time to progress; DFS, disease-free survival; TTR, time to recurrence; N.A. not available.

## The Regulatory Mechanism Underlying E-Twenty-Six Family in Hepatocellular Carcinoma

Post-translational modifications are vital for these factors, such as phosphorylation. For instance, the Ras/Raf/MEK/ERK signaling cascade, one of the most important dysregulated pathways in HCC, plays a pivotal role in regulating the ETS superfamily. Activated ERK1/2 in the nucleus can nearly phosphorylate all the oncogenic ETS factors *in vitro* (13). ERK could phosphorylate ETS1 at T38 and S41 residues in various cell types, necessary for ETS1 to activate migration genes of epithelial cells modulated by Ras (51). Phosphorylation of ELK1 at Ser383 induced by sustained ERK activation promotes apoptotic cell death *via* upregulating death receptor 5 expression (52). What is more, ELK1 phosphorylation could be impeded by MDM2 binding protein *via* inhibiting nuclear translocation of phosphorylated ERK (53). It is worthy to note that ERK could indirectly promote ETS expression by regulating CIC, a tumor suppressor in various cancers, including HCC. CIC repression on PEA3 sub-group genes (ETV1, ETV4, and ETV5) is relieved following ERK phosphorylation (54).

Noteworthy, there are specific microRNAs (miRNAs) that participate in the regulation of ETS factors expression. MiR-324-5p negatively regulates ETS1 expression, thus inhibiting the modulation of MMP2 and MMP9 by ETS1 (55). Overexpression of miR-193b and miR-129-5p could, respectively, lead to decreased mRNA and protein levels of ETS1 *via* directly targeting its 3′-UTR (56, 57).

It has been recently shown that ETS1 could be modified by m6A in HCC, mediated by Wilms tumor 1-associated protein (WTAP) (34). WTAP, a vital component of the methyltransferase complex, increases m6A modification of ETS1 mRNA by interfering with the connection between HuR protein and ETS1 mRNA, and thus represses ETS1 expression.

Another interesting mechanism is that ETS factors can obtain transcriptional binding motif and actively bind to the region, provided by cis-acting mutations in the telomerase reverse transcriptase (TERT) promoter (6, 58). Somatic mutations of TERT promoter have been observed in some cancers, such as melanoma (71%) and HCC (60%) (59). It is one of the most common genetic alterations in HCC. Unfortunately, TERT is not clinically actionable in HCC at present (2, 60). Sequencing the promoter region of TERT in 305 HCC samples, Nault et al. found that mutations located at -124bp and -146bp from the ATG start site, consisting of G to A or G to T substitutions, generated typical ETS-binding motifs (61). GABAα could be selectively recruited to the mutant TERT core promoter (CCGGAA) in HepG2 cells harboring G228A mutation, activating TERT gene expression (58). In HCC, telomerase activation is critical in driving hepatocarcinogenesis. Recently, another novel cis-activating mechanism has been revealed: binding to the chimeric HBV EnhI at the TERT promoter, ELF4 could achieve TERT activation in TERT HBV-integrated HCC (62).

## Effects of E-Twenty-Six Family on Hepatocellular Carcinoma Cells

**Figure 1:**
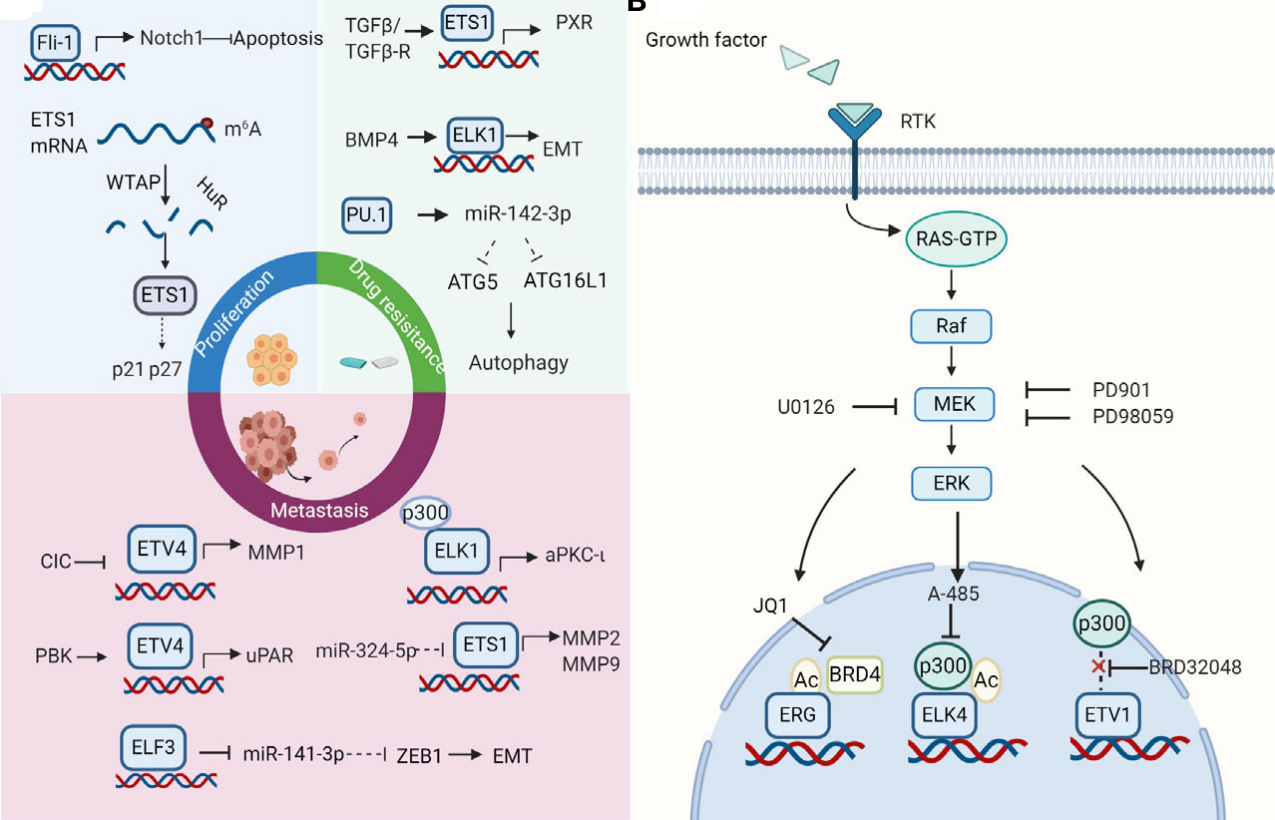
Roles and therapeutic strategies of the E-twenty-six (ETS) family. **(A)** Effects of ETS family on hepatocellular carcinoma (HCC) cells. ETS factors take part in cell proliferation, invasion, metastasis, and drug resistance in HCC. Selected examples of ETS factors and their upstream factors or genomic targets are shown. Diagram is adapted from reference (63). **(B)** ETS transcription factors as therapeutic targets. The strategies for targeting ETS factors include breaking its association with protein partners, inhibiting its regulatory pathways, using its small molecule inhibitors. The strategy of combination therapy to target the ETS family in cancers appears to be a promising way for future research. Created with BioRender.com.

As cancer cells acquire a range of capabilities, such as sustained proliferation, resisting cell death, inducing angiogenesis, invasion, metastasis, and immune escape, they start to get out of control in human bodies (63). This process is brought by the changes in gene structure and function of cancer cells. Deregulation of ETS factors expression has been found to influence cellular proliferation, epithelial-mesenchymal transition (EMT), invasion, metastasis, and drug resistance of HCC cells (**Figure 1A**).

## E-Twenty-Six Family and Hepatocellular Carcinoma Cell Proliferation

Several studies have reported roles that ETS genes play in HCC proliferation. Chen et al. found that PDEF repressed cell proliferation by regulating their apoptosis and cell cycle in HCC (39). Moreover, Fli-1 inhibits HCC cell apoptosis and facilitates cell proliferation by regulating the levels of Bax and Bacl2 *via* the Notch1 pathway (37). EHF is mainly located in the cytoplasm and suppresses HepG2 cell growth both *in vitro* and *in vivo* (46). Interestingly, the different expression of ETS1 in the nucleus and cytoplasm may exhibit different roles in HCC cell proliferation. The elevated expression of ETS1 in the nucleus induces expression of HK1 and PFKFB3 and rescues the decrease of HCC cell proliferation induced by miR-139-5p in SK-Hep-1 and SMMC-7721 cells, indicating that ETS1 probably plays a carcinogenic role in HCC (35). On the contrary, decreased expression of ETS1 in cytoplasm mediated by WTAP leads to down-regulation of p21 and p27 and promotes HCC cell proliferation (34).

## E-Twenty-Six Family and Hepatocellular Carcinoma Cell Invasion and Metastasis

Studies have demonstrated a connection between high ETS expression and increased HCC cell invasion and metastasis. ETS1 could upregulate MMP2, MMP7, and MMP9 expression to enhance HCC cell invasive and metastatic capacity (55, 64). ETS2 promotes cell invasion by interacting with MLL to transactivate MMP1 and MMP3 (65). ELF3 represses E-cadherin and promotes EMT in HCC cells *via* suppressing miR-141-3p, thereby activating ZEB1 (38). ELK1 promotes invasion and EMT in HCC cells *via* upregulating aPKC-ι expression (66). Knocking down ETV4 completely blocks the facilitation of cell invasion and migration mediated by CIC deficiency (67). ETV4 can also modulate cell invasion and migration by directly binding to the core region of the uPAR promoter (68). Meanwhile, PDZ-binding kinase (PBK) could promote the binding of ETV4 to uPAR promoter. Combined, these studies indicate that targeting ETS factors could be a promising therapeutic strategy to treat HCC, especially in its metastatic forms.

## E-Twenty-Six Family and Hepatocellular Carcinoma Cell Drug Resistance

Recently, some ETS factors have been recognized to be functional players in drug resistance. ETS1 could interact with the pregnane X receptor (PXR) to promote its accumulation in the nucleus, thus accelerating sorafenib clearance from hepatoma cells. In the meantime, ETS1 could improve the sorafenib-resistance of HCC *in vivo* tumor models (36). Another study further explored the upstream regulation mechanism of ETS1 in drug resistance (50). TGFβ1/ERK could promote drug resistance at the late stages of HCC. TGF-β pathway enhances the binding of ETS1 to the PXR promoter. Moreover, ERK inhibitor effectively blocks TGFβ - induced PXR expression. Furthermore, evidence suggests that acquired chemoresistance is closely related to EMT (69). ELK1 could mediate BMP4-induced oxaliplatin resistance in HCC, attributed to EMT initiation through E-cadherin (70). Blocking MEK/ERK/ELK1 could weaken EMT and improve the sensitivity of oxaliplatin (70). Finally, the cytoprotection of autophagy is another drug resistance mechanism, enabling cells to adapt to environmental stress and protect HCC cells treated with sorafenib from death. PU.1/miR-142-3p axis could target ATG5 and ATG16L1, two key autophagy-related proteins, to suppress protective autophagy and enhance apoptosis, thereby improving the sensitivity of HCC cells to sorafenib (40).

## The Therapeutic Promise in Targeting E-Twenty-Six Factors in Hepatocellular Carcinoma

Transcription factors were long considered to be “undruggable” targets as their potential for off-target effects and insufficient delivery within the cell. Nevertheless, multiple attempts have been made to target ETS factors directly or indirectly, some of which appear promising. Moreover, interest in combination therapies is rapidly increasing as it may improve the outcomes in the front-line setting. Studies above suggest that ETS proteins may be a potential therapeutic target for HCC. Although the therapeutic strategy of targeting ETS factors in HCC has not been thoroughly studied, studies in other cancers may provide some ideas for the treatment of HCC (**Figure 1B**).

One way of targeting ETS factors is breaking interaction with their transcriptional co-factors. For example, CBP/p300 are major histone acetyltransferases (HATs) that could interact with and acetylate ETS transcription factors (21). A series of CBP/p300 inhibitors could inhibit the expression and activity of ETS members effectively. CBP30, a small‐molecule inhibitor of EP300, which is the vital constituent of the CBP/p300 complex, markedly inhibits the expression of ETV4 in HCC cells (71). A-485, an inhibitor of CBP/p300, could decrease the level of acetylated ELK4 and limit its transcriptional activity (72). Besides, acetylated ETS factors could be recognized by BET proteins. These BET proteins build transcriptional regulatory complexes by recognizing acetylated lysine and accumulating transcriptional activity elements (73). BET inhibitors, some of which have been in clinical trials, could suppress the transcriptional activity and expression of ETS factors (74, 75). For instance, transcriptional activation of ELK4 target genes could be inhibited by JQ1, a BET inhibitor, *via* disrupting the interaction between ELK4 and BRD2 in colorectal cancer (72). JQ1 could inhibit the function of acetylated ERG, which recruits BRD4 to promote transcriptional activation (76). Treatment with JQ1 or OTX015 represses the expression of ETV4 in HCC (71). However, the mechanism between BRD4 and ETV4 in HCC needs further research. Notably, a combination of A-485 and JQ1 has synergistic anti-tumor effects (77).

Alternatively, the pathway which regulates ETS factors could be targeted. For example, MEK/ERK inhibitor PD98059 could effectively decrease the expression of p-ETS1 in HCC cells treated with recombinant TGF-β (50). So far, numerous MEK inhibitors have been developed, but they have limited clinical benefits in solid tumors, including advanced HCC, as single-agent therapies. Researchers began to explore its efficacy as a combination drug. MEK and KIT inhibitors impede gastrointestinal stromal tumor (GIST) growth by interrupting the KIT–ETV1-positive feedback circuit in GIST (78). Moreover, MEK inhibitor U0126 exhibits a synergetic effect with BET inhibitor JQ1 *via* ELK4 activity inhibition in colorectal cancer (72). Besides, the combination of MEK inhibitor PD901 and JQ1 has a more significant inhibition on the expression of several ETS factors (ELK3, ETV1, ETV4, ETV5) than MEK inhibitor alone in lung cancer (79).

Encouragingly, plenty of small molecule inhibitors targeting ETS proteins such as VPC‐18005, YK‐4‐279 have been developed during the past few years and play a role in cell lines with chromosomal translocation (80). Notably, BRD32048, a tricyclic compound, can also be applied for ETV1-amplified cell lines. BRD32048 inhibits acetylation of ETV1 mediated by p300, thus facilitating its degradation. However, it is not clear whether BRD32048 blocks ETV1 residues K33 acetylation or affects some protein-protein interactions. Moreover, binding to ETV1 directly, BRD32048 suppresses the transcriptional activity of ETV1 on the MMP1 promoter to impede cancer cell invasion and proliferation (81). In addition, structure identification has determined protein 3D structures of many ETS factors, which helps develop structure‐based drugs (80). With the development of new technology and methods such as computer‐aided drug design, it will be possible to find more ETS inhibitors (13). Combinations of these ETS inhibitors and other types of inhibitors mentioned above could provide more strategies for HCC.

## Conclusion

HCC is a highly invasive tumor, which is the major cause of the high recurrence rate after surgical resection. For patients with an unresectable tumor or at an advanced stage, drug resistance of tumor often leads to treatment failure. In this respect, identifying potential biomarkers and molecular mediators would help predict the prognosis of this malignancy.

The above studies have shown that the aberrant expression of some ETS factors are related to HCC, suggesting that they could be potential prognostic markers for HCC. ETS family regulates HCC cells, including but not limited to cell proliferation, invasion, metastasis, and drug resistance. Therefore, it is worthwhile to consider some ETS factors as potential targets of HCC. Furthermore, ETS factors expression and activity could be regulated at the transcriptional, post-transcriptional, and post-translational levels. The strategies for targeting ETS can be divided into (1) breaking its association with protein partners, (2) blocking its regulatory pathways, (3) directly inhibiting themselves. Epigenetic inhibitors such as HAT inhibitors and BET inhibitors repress the expression and activity of ETS factors and have enticing suppressive effects in cancers. However, the underlying mechanisms need to be further elucidated. Moreover, the Ras/Raf/MEK/ERK pathway is the most critical regulatory pathway for ETS factors, the inhibitors of which have limited efficacy as a single agent. Evidence suggests that the combination therapy of these inhibitors with additional agents will be of better effects. Also, small-molecule inhibitors targeting ETS proteins are in development, which is likely to serve as one component of combination therapy.

The studies about the ETS family in HCC are currently limited. Recently, emerging evidence has suggested that besides HCC, ETS factors function as important regulators in other gastrointestinal cancers. For instance, overexpression of ETV4, ELF3, and ETV5 facilitates tumor angiogenesis, growth, invasion and metastasis, and indicates poor prognosis (82–85). Besides, ETS1 could regulate cell viability *via* interfering glycolysis in pancreatic cancer (86); increased expression of ETS2 promotes drug resistance in colorectal cancer (87). Interestingly, unlike its tumor-suppressive role in colorectal cancer and HCC, PDEF is overexpressed in gastric cancer and promotes gastric cancer cell proliferation by regulating FoxM1 expression through a positive feedback loop (39, 88, 89). In conclusion, ETS family may offer vast potential for developing novel therapeutic strategies. With the deepness of research, it will be promising that targeting ETS factors is applied in HCC and other cancers.

## Author Contributions

TZ, DL, YW, and MS performed the literature search and manuscript drafting. LX supervised and revised the manuscript. All authors contributed to the article and approved the submitted version.

## Funding

Research was supported by grants from the National Key Research and Development Program of China 2018YFC1312103 (L.X.), National Natural Science Foundation of China No. 81972237 (L.X.), and No. 81772623 (L.X.).

## Conflict of Interest

The authors declare that the research was conducted in the absence of any commercial or financial relationships that could be construed as a potential conflict of interest.
